# Exploring the Barriers and Motivators to Dietary Adherence among Caregivers of Children with Disorders of Amino Acid Metabolism (AAMDs): A Qualitative Study

**DOI:** 10.3390/nu14122535

**Published:** 2022-06-18

**Authors:** Jing Ying Lim, Roslee Rajikan, Noh Amit, Nazlena Mohamad Ali, Haslina Abdul Hamid, Huey Yin Leong, Maslina Mohamad, Bi Qi Koh, Aini Musa

**Affiliations:** 1 Dietetics Program, Centre of Healthy Aging and Wellness (H-Care), Faculty of Health Sciences, Universiti Kebangsaan Malaysia, Kuala Lumpur 50300, Malaysia; jingying1610@gmail.com; 2 Dietetics Department, Faculty of Medicine and Health Sciences, Universiti Putra Malaysia, Serdang 43400, Malaysia; 3 Clinical Psychology and Behavioral Health Program, Center for Community Health Studies (ReaCH), Faculty of Health Sciences, Universiti Kebangsaan Malaysia, Kuala Lumpur 50300, Malaysia; nohamit@ukm.edu.my; 4 Institute of IR4.0 (IIR4.0), Universiti Kebangsaan Malaysia, Bangi 43600, Malaysia; nazlena.ali@ukm.edu.my; 5 Dietetics Program, Center for Community Health Studies (ReaCH), Faculty of Health Sciences, Universiti Kebangsaan Malaysia, Kuala Lumpur 50300, Malaysia; haslina86@ukm.edu.my; 6 Genetics Department, Hospital Kuala Lumpur, Kuala Lumpur 50300, Malaysia; hueyyinleong@yahoo.com; 7 Dietetics & Food Service Department, Hospital Kuala Lumpur, Kuala Lumpur 50300, Malaysia; maslina.affendi@gmail.com (M.M.); biqikoh@gmail.com (B.Q.K.); 8 Dietetics & Food Service Department, National Cancer Institutes, Putrajaya 62250, Malaysia; a2_88@hotmail.com

**Keywords:** barriers, motivators, dietary adherence, disorders of amino acid metabolism

## Abstract

Dietary intervention is generally accepted as the mainstay of treatment for patients with disorders of amino acid metabolism (AAMDs). However, dietary adherence to a low-protein diet is always reported as a common challenge among these patients. This study explored the perception of barriers and motivators to dietary adherence among caregivers of AAMD patients in Malaysia. Twenty caregivers of children with AAMDs receiving ongoing treatment at the genetic clinic participated in an online focus group discussion from November to December 2021. Findings showed a total of five interrelated main themes identified from focus group discussion (FGD) exploring parents’ experiences related to the management of their child’s daily diet. The barriers to dietary adherence were burden of dietary treatment, diet and dietary behavior, parenting challenges, limited knowledge related to dietary treatment, and challenges in healthcare system delivery. Key factors facilitating good dietary adherence include good knowledge of dietary treatment, parental coping strategies, social coping, and dietary behavior. In conclusion, despite the existence of several barriers to the implementation of dietary treatment, caregivers managed to use a wide range of coping strategies to overcome some, if not all, of the challenges. The important next step is to develop, in conjunction with multidisciplinary healthcare professionals, feasible implementation strategies that could address these barriers and at the same time improve the quality of life of caregivers.

## 1. Introduction

Disorders of amino acid metabolism (AAMDs) are among the groups of inherited metabolic disorders (IMDs) that lead to the accumulation of toxic substrates in the blood, urine, and body tissues, including the brain [1,2]. This condition is attributed to the deficiency of an enzyme, its cofactor, or a transporter in the metabolic pathways of amino acids. According to the International Classification of Inborn Metabolic Disorders [3], AAMDs can be further classified into a variety of subgroups that include urea cycle disorders (UCDs), organic acidurias (OAs), and aminoacidopathies (AAs). Globally, the prevalence of AAMDs at birth is reported as 26.31 per 100,000 live births [4]. In Malaysia, Maple Syrup Urine Disease (MSUD) is reported as the most common subclass of AAMDs, especially among the Malay population, with 1 per 9720 live births based on a newborn screening program [5]. In 2020, it was reported that 33 patients were diagnosed with MSUD [2].

It is now well established from a variety of studies that individualized dietary therapy plays an important role in the treatment of AAMD, as it is characterized by a disruption of the metabolism of amino acids, which are the main constituents of the daily diet [6,7]. The current treatment for this group of disorders includes (a) the provision of a suitable protein substitute free of the offending substrate; (b) a lifelong protein restriction of natural protein intake through the exclusion of all high protein food, such as eggs, milk, cheese, meat, poultry, fish, dried beans, and legumes; and (c) the provision of special low-protein food to meet the energy requirement [8,9]. The daily requirement and allocation of natural protein for each individual are calculated and titrated according to the longitudinal metabolic control and the plasma essential amino acid level over time [10]. As such, a close follow-up with a metabolic pediatrician and metabolic dietician is necessary. Patient adherence to dietary treatment is paramount to maintain blood amino acid levels within a target range, which is not associated with neurotoxicity, to achieve good nutritional status, and to prevent endogenous catabolism [11,12,13].

Despite regular follow-up with multidisciplinary healthcare professionals, non-adherence to dietary treatment remains the main challenge in the management of patients with AAMDs [14,15]. Furthermore, it is revealed that dietary adherence and optimum metabolic control decline with age, particularly in patients approaching adolescence and adulthood [16]. Several studies have documented that phenylketonuria (PKU) patients with weak dietary compliance exhibited poorer development in terms of intelligence quotient and schooling performance, as well as a higher frequency of complications, such as mental retardation, attention deficit hyperactivity disorder, cerebral palsy, epilepsy, and osteopenia [17,18]. Furthermore, recent evidence has suggested that ataxia, the loss of coordination and emesis, associated with hyperleucinemic episodes in MSUD patients, is triggered by non-compliance with dietary recommendations, which include excess natural protein intake, and reduced caloric intake, leading to increased catabolism [19].

The non-adherence to dietary therapy might be attributed to the extremely restrictive nature of the dietary treatment. Adherence to a rigorous dietary regime, such as a vegetarian-like diet, was found to be one of the biggest challenges for the patient and the entire family, particularly at the early stages of diagnosis [20]. Difficulties were encountered during the planning and preparation of a low-protein diet, as this requires knowledge of food contents and recipes, as well as continuous measurements of ingredients [21]. To compound matters, the temptation to overindulge in prohibited foods can be challenging for a patient, considering the availability of a wide variety of protein-rich foods [22,23]. A qualitative study involving PKU adolescents in Australia revealed that they faced difficulties trying to incorporate the required diet into their school routine due to a poor understanding of their situation among their teachers and friends [23].

However, social support from friends and family, as well as healthcare providers, such as metabolic pediatricians and dieticians, was identified as a motivator for dietary adherence [23,24]. Furthermore, awareness of the importance of maintaining good metabolic control and the desire to preserve important relationships motivated patients to adhere to a strict dietary regime, as they associated the consequences of dietary non-adherence with negative effects on their social interactions [23,24].

Of late, researchers have increasingly utilized the focus group discussion (FGD) approach to elicit the perception of patients with regard to adherence to a dietary treatment [25]. This method allows researchers to probe areas that are inaccessible through quantitative research and better understand the experiences and perspectives of caregivers, which are essential for the development of effective dietary strategies to support patients and their families with regard to dietary adherence [26].

To date, patient experiences regarding dietary management have been ascertained through the broader experiences regarding the management and treatment of disease, as well as its impact on child and family life, while neglecting the issue of dietary treatment, where diet was only a minor part of the study [20,27]. Moreover, the current qualitative study approach focuses solely on investigations regarding the factors affecting dietary adherence among the PKU population [22,23] while disregarding other various types of amino acid metabolism disorders. Furthermore, the emphasis of these studies was on PKU adolescents and adults rather than caregivers of patients. It is assumed that the clinical manifestations of different disorders vary from moderate to severe neuropsychological problems. As such, the inclusion of caregivers watching over patients with different types of AAMDs will provide a clearer picture of the factors affecting dietary adherence. This qualitative study aims to address this gap and provides a better understanding of the barriers and motivators, with a view to better define the potential interventions that may improve dietary adherence among AAMD patients.

## 2. Materials and Methods

### 2.1. Theoretical Framework

Phenomenology was used as a qualitative approach in this study. This approach was used to understand the context of the experience in managing daily diet, including the barriers and motivators among the caregivers. During the discussion, the caregivers expressed how they coped with the dietary restriction at home and within the social community including schooling and traveling into a phenomenon [28].

### 2.2. Participants’ Selection and Recruitments

The participants of this study included caregivers of patients diagnosed with AAMDs. In brief, the patients are receiving active treatment at the genetic clinic, Hospital Kuala Lumpur (HKL), which is the national referral center for IMD patients. Caregivers aged above 18-year-old with patients aged between 6 months - 18-year-old were included in this study. Patients who had not attended an appointment in the genetic clinic for the last 2 years since the date of data collection were excluded. Purposive sampling with the maximum variation strategy was used in this study to achieve a sample of caregivers of children of different races, age groups, clinical conditions, and types of AAMDs. Since metabolic disorders are a heterogeneous group of disorders, employing this strategy enabled the researcher to identify essential and variable barriers faced in dietary adherence experienced by caregivers within a varied context [29].

According to Guetterman [30], a sample size of 25 participants was anticipated to reach data saturation in the field of health science. Hence, a total of 30 caregivers were approached either face-to-face or via phone call, taking into consideration the possibility of non-participation. The phone number of the caregivers were obtained from the metabolic dietitian who kept a personal record of the caregivers. All the caregivers were inquired about internet access and their preferred online video conference software. At the same time, they were given a list of choices for the day and time in which the discussion will be held based on their availability. Caregivers who were interested to participate in this study were asked to fill in and sign the informed consent form. Patients were contacted with a maximum of three attempts. The reasons for refusal to participate included limited access to internet connection, time constraints, and not interested in the discussion. The study was approved by the Medical Research Ethics Committee (MREC) (NMRR-21-1845-6108) and the Secretariat of Research Ethics Committee, Univerisiti Kebangsaan Malaysia (JEPUKM-615 2021-765).

### 2.3. Study Settings and Data Collection Procedures

This study was conducted by way of a virtual platform, with the use of 2 forms of video conferencing software: Google Meet and Zoom, from November to December, 2021, in view of the advent of the COVID-19 pandemic. The use of two different platforms served to accommodate participant preferences. The virtual focus group discussion (FGD) was conducted by 2 researchers: one to moderate the discussion, and the other to take down notes. The female moderator, the first author, is a postgraduate student researcher with a Bachelor’s degree in dietetics, trained in qualitative research. The link for participation in the meeting was provided to the caregivers one day prior to the FGD. The caregivers were reminded of the date and time of the discussion.

The discussion guide was established to ensure consistency across the various focus groups. The questions covered a broad scope of literature related to AAMDs [20], which were revised through discussions among researchers with expertise in qualitative methods (Table 1). During the FGD, the moderator employed suitable probing questions to elicit an in-depth description of the participants’ experiences. Each FGD session, which lasted between 60 and 90 min, was audio and video recorded. At the end of the discussion, overall summarization of important points was made.

### 2.4. Data Coding and Analysis

The Consolidated Criteria for Reporting Qualitative Research (COREQ) framework was used to guide the reporting of the findings [31]. The ATLAS.Ti version 10 software (ATLAS.ti Scientific Software Development GmbH, Berlin, Germany)was used for data management and analysis. Data analysis was carried out using thematic analysis [32], employing the steps as proposed by the Framework Method [33]. In our analysis, a hybrid approach of inductive and deductive thematic analysis was employed [34]. This approach complemented the research questions by allowing the phenomenon of barriers and motivators of dietary treatment to be integral to the process of deductive thematic analysis while allowing for themes to be driven directly from the data set [34]. A coding scheme that included criteria used to classify and interpret empirical observations was applied as a means of organizing text for subsequent interpretation of the transcript [35]. At the same time, a previous study [10], which outlined the factors affecting diet adherence, was used as a framework (deductive approach) to generate codes linking to the research objectives, which are the barriers and motivators of dietary adherence. The process of coding was carried out by the student researcher, and three other researchers with expertise in dietetics and nutrition, health psychology, and information technology were tasked to check the initial contents based on the research objectives.

The data collection and analysis were carried out concurrently so that new emerging themes could be queried or probed further in the subsequent FGD. A Microsoft Excel spreadsheet was used to generate a matrix; the data were ‘charted’ into the matrix, in which the codes were charted in columns, and the cases were charted in rows [35]. Subsequently, the codes were sorted into potential subthemes, and the relevant subthemes were combined into meaningful themes, which were then clearly defined. All the codes, themes, and subthemes were reviewed and refined among the research team members until a mutual agreement was achieved [32].

### 2.5. Trustworthiness

Multiple approaches were used to enhance the rigor of data analysis. Firstly, member checking was carried out by sending back the transcripts, which were all verbatim, to the participants for checking. Next, triangulation was used by involving at least two related data sources, such as official records, observation, and field notes. The transcript was checked against the official records, such as the participants’ diet recall to determine whether any variation emerges from the two data sources. The ethical approval to obtain the official record was acquired from a previous retrospective cross-sectional study conducted to determine the nutritional status of the patients. Besides that, observation was used during the process of the participants’ recruitment throughout a previous cross-sectional study. The caregiver’s feeding practices and the children’s dietary behavior of the children were observed at the genetic clinic, Hospital Kuala Lumpur (HKL). Lastly, the transcript was checked against the field notes made by another research enumerator to ensure the validity of the data [36]. Besides that, peer debriefing was applied where biweekly meetings were conducted between the student researcher and her academic supervisors who are skilled qualitative researchers with expertise in dietetics and nutrition, clinical psychology, and information technology (IT) on the research methodology, data analysis, and interpretations continuously throughout the research process [36].

## 3. Results

### 3.1. Sociodemographic Characteristics

A total of 20 caregivers from 18 families signed informed consent and took part in five virtual FGDs. Although there was refusal to participate in the study, data saturation was reached at the 5th focus group [37] and at the 20th participant. Each group comprised three to five participants and ranged from 1 h to 1 h and 30 min. Table 2 shows the participants’ sociodemographic characteristics. The majority of the participants were female (90%), Malay (65%), working (55%), and possessed at least a diploma or degree-level education (60%). Most of the families had children with aminoacidopathies (AAs) (38.8%), followed by organic acidurias (OAs) (33.3%) and urea cycle disorders (UCDs) (27.8%).

### 3.2. Barriers to Dietary Treatment

**Figure 1:**
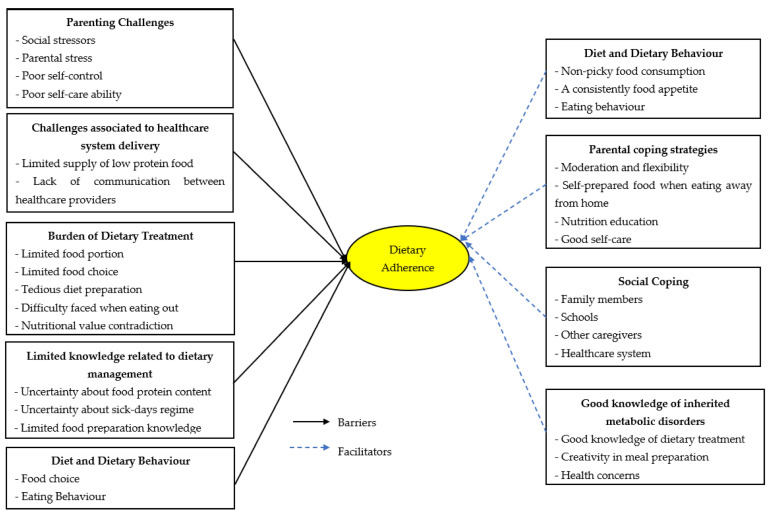
Barriers and facilitators to dietary adherence among AAMDs patients.

In this study, five themes emerge as barriers to dietary adherence. Namely, these are the burden associated with dietary treatment, diet and dietary behavior, parenting challenges, limited knowledge regarding dietary management, and challenges associated with healthcare system delivery. Selected quotations that exemplify each theme and subtheme are provided as Supplementary Data (Table S1). Figure 1 summarizes the barriers and motivators to dietary adherence among AAMD patients.

#### 3.2.1. Burden of Dietary Treatment

The dietary treatment for AAMD patients includes the restriction of natural protein in the daily diet to a limited amount. This poses a challenge for caregivers and children under their care in terms of food selection when eating out, making decisions on daily food choices and portions, and the preparation of daily meals. Moreover, a diet low in natural protein is viewed as contradictory to the ‘healthy eating’ concept. The dietary treatment during sick days, which includes omitting the consumption of natural protein and replacing it with the consumption of a medical formula, is also considered a challenge.

##### Tedious Food Preparation

One of the most frequently perceived burdens was the tedious and complex process associated with the preparation of a low-natural-protein diet. In order to deliver an accurate amount of natural protein in each meal, the caregivers were required to weigh and calculate every food item before serving, which is perceived as time-consuming and stressful. Furthermore, caregivers faced difficulties in calculating the accurate amount of protein, especially when it came to new food items. Some caregivers experienced problems during the preparation of unfamiliar low-protein food for their children.

##### Difficulty Faced When Eating Out

Caregivers also faced difficulties deciding on a suitable low-protein diet when eating out, as most of the foods on the menu in restaurants and school canteens are prohibited to the children under their care.

##### Limited Food Portion

Issues related to patient dissatisfaction regarding the limited food portion served were also raised during the discussion session. The food portion needed to be strictly controlled so as not to exceed the prescribed natural protein intake. It is notable that the majority of cereal-based food comes with a small amount of natural protein.

##### Limited Food Choices

A variety of perspectives were expressed in terms of food choices. Among the most daunting challenges was the need to completely exclude high-protein foods, such as meat, eggs, milk, and cheese, as well as their products, from the patient’s diet. This drastically reduced their food choices. However, a problem also arose when it came to snacks such as chocolate, ice cream, cookies, and cakes, which come with prohibited ingredients but are usually highly favored by children. Another issue was the limited variety of special low-protein food (zero protein) available in this country.

##### Nutritional Value Contradiction

Concerns were raised regarding the nutritional value of low-protein products, which are high in carbohydrates and sugar, as their consumption is in contradiction to the ‘healthy eating’ concept.

##### Challenges during Sick Day

The need to omit the consumption of intact protein (natural protein) during sick days was one of the challenges mentioned by the caregivers, as the child under their care still craved prohibited natural protein food during this period.

#### 3.2.2. Diet and Dietary Behavior

The diet and dietary behavior of AAMD children were also identified by the caregivers as major challenges. This broad category was further divided into two subthemes: food choice and eating behavior. The results from our investigation indicated that some children were exceedingly selective about certain food items (such as vegetables) and special low-protein food. Eating behavior refers to the actual act of consumption, including eating habits, portions, frequency, and disordered eating symptoms [38].

##### Food Choice

Food preferences varied among AAMD children. While some are content with the same diet routine, and shy away from trying something new, others are not in favor of consuming low-protein products.

##### Eating Behavior

Several issues related to the children’s eating behavior were reported. Among them were the issues of vomiting due to overeating, a prolonged mealtime duration due to poor self-feeding and sensory processing skills, and undesirable weight gain due to overeating.

#### 3.2.3. Parenting Challenges

The imposition of strict restrictions poses many challenges for parents and children. These challenges included the stress encountered during meals with peers, a poor understanding of the conditions that need to be complied with, and personal factors, such as poor self-control with regard to food portions and poor self-care ability due to emotional problems.

##### Social Stress

Some caregivers mentioned that their children experienced feelings of discomfort during certain social situations such as when eating with their peers or siblings. While their craving to try the food consumed by their peers or siblings increases as they grow older, their condition prohibits them from doing so.

##### Parental Stress

During this study, it was uncovered that the dearth of pre-school establishments willing to accommodate the dietary needs of children with AAMD was among the sources of stress for their parents. Furthermore, parental stress also occurred when there was a demand from the parents for their child to consume more food and metabolic formula, but the child was unable to take them and vomited them out. In addition to that, the awareness and understanding of the dietary requirement, clinical signs, and symptoms related to metabolic disorders seem to be poor and limited among the public, resulting in parental stress.

##### Poor Self-Control

Poor self-control often took over when children were left without parental supervision. In such a situation, children may consume more food than the recommended portion, as well as skip the consumption of their metabolic formula, in favor of consuming food prohibited to them.

##### Poor Self-Care Ability

Poor self-care ability of children with AAMD can be attributed to the impact of their condition on their neuropsychological profile. Some caregivers reported that the children under their care were not up to the mark when it came to managing their own daily activities. This included the preparation of their metabolic formula, which often requires the assistance of a teacher.

#### 3.2.4. Limited Knowledge Regarding Dietary Management

Limited knowledge was observed to be among the major barriers to good dietary management. This issue was mainly associated with uncertainty about the nutritional value of a certain food, poor food preparation skills, and limited knowledge about dietary management for sick days, as well as for a metabolic crisis.

##### Uncertainty about Food Protein Content

Several participants were generally uncertain about the accurate protein content in food. This led to less variation in the daily diet of the children, as the caregivers were reluctant to introduce food without verifying its protein content. Uncertainty in this area also led to underestimation of the daily natural protein intake.

##### Limited Food Preparation Knowledge

The limited knowledge and lack of creativity with regard to low-protein food preparation led to food boredom among children and consequently the consumption of less (inadequate) food.

##### Uncertainty about Sick-Day Regime

Several caregivers felt challenged when it came to dietary management in a sick-day situation. In our study context, the challenges include solutions to a metabolic crisis, as well as remedies to a lack of appetite and vomiting, which can lead to an inadequate intake of natural protein.

#### 3.2.5. Challenges Associated with Healthcare System Delivery

A few participants expressed their concerns regarding the current healthcare system. To begin with, they were of the opinion that the hospital’s provision of low-protein food is exceedingly limited.

Furthermore, the lack of communication between healthcare providers was looked upon as a hindrance to the delivery of good service and information.

### 3.3. Motivators of Dietary Treatment 

The key factors motivating good dietary adherence include good knowledge of dietary treatment, parental coping strategies, social coping skills, and good dietary behaviors. Selected quotations that exemplify each theme are provided in the Supplementary Data (Table S2).

#### 3.3.1. Good Knowledge of Inherited Metabolic Disorders

Generally, the participants demonstrated good knowledge of the risk factors, complications, and dietary treatments related to metabolic disorders in terms of daily diet planning and protein calculation, as well as specific management abilities to cope with sick days.

##### Good Knowledge of Dietary Treatment

Most of the caregivers were able to identify natural food sources low in protein content and estimated the required food portion visually or with the use of household measurements. Their efficiency in this area improved with experience. Generally, the caregivers were aware of the children’s natural protein requirements and prepared their daily diet according to the directives from healthcare professionals. Several caregivers were also familiar with the sick-day regime and were able to execute remedial steps such as increasing the dosage of branched-chain amino acid (BCAA) supplementation and restricting the consumption of solid food.

##### Creativity in Meal Preparation

Some caregivers displayed creativity with regard to the preparation of the children’s daily diet. This included the use of a wide variety of low-protein ingredients, such as corn starch instead of normal flour, so that the children would not be bored by the same food every day.

##### Health Concerns

Most of the caregivers emphasized the importance of taking natural protein within the recommended intake to prevent complications such as hospitalization, neurological impairment, and disability. A few participants emphasized the importance of an accurate protein intake for the children to ensure their proper development. Aside from the focus on natural protein, the significance of consuming a prescribed metabolic formula to ensure the child’s adequate energy intake was also mentioned.

#### 3.3.2. Parental Coping Strategies

Apparently, caregivers managed the complexity associated with restricted diets and treatment regimens through the employment of various coping strategies. These include flexibility and moderation with regard to the child’s diet, self-preparation of food when eating away from home, education on nutrition, and equipping the child with effective self-care attributes.

##### Moderation and Flexibility

Caregivers practiced moderation by reducing the normal portion of prohibited food to satisfy the child’s craving for such food. Caregivers also reduced the normal portion of certain food in situations where they were uncertain about its protein content level. As for flexibility, this comes into play with adjustments to the daily meal plan to counteract the effects of overconsumption or inadequate consumption of certain food.

##### Self-Prepared Food when Eating away from Home

To cope with the challenge of providing the children with suitable food, when eating away from home, the majority of caregivers equipped themselves with a cooker and raw ingredients and self-prepared the meal for the children or prepared the children’s food and the metabolic formula at home for consumption during break time at school.

##### Nutrition Education

Caregivers also provided education for their children on the complications associated with the consumption of ‘prohibited’ food and coached them on the proper selection of food when eating away from home.

##### Effective Self-Care

As children grow in maturity, they are expected to take on more responsibilities when it comes to their diet treatment. These responsibilities include the preparation of their metabolic formula and the selection of suitable food when eating out.

#### 3.3.3. Social Support

In the context of this study, social support refers to the emotional or instrumental support extended to caregivers by the community, which includes family members, other caregivers, teachers in schools, and operators in the healthcare system.

##### The Healthcare System

In the context of the dietary treatment process, the healthcare system represents the main support of caregivers. Most caregivers considered the printed nutritional booklet provided by the dietitian as their main source of information. Several participants stated that they would consult the dietitian and/or doctor when in doubt about issues relating to food or the metabolic formula. Meanwhile, several caregivers were in favor of dietetic counseling sessions as a means of improving their knowledge of nutrition-related issues.

##### Schools

Caregivers seek the support of teachers when the children under their care begin schooling. By adhering to the instructions from parents, teachers ensure that the children afflicted with AAMD are provided with an appropriate diet while at school. Teachers also carry the responsibility of preparing the metabolic formula for children who are self-care impaired.

##### Family Members

The involvement of family members (such as the patient’s grandparents and siblings) with regard to the patient’s food consumption can go a long way towards ensuring that their diet complies with parental instructions.

##### Subtheme 4: Other Caregivers

The information and experiences shared by peers can serve to enhance the quality of care delivered by a caregiver.

#### 3.3.4. Diet and Dietary Behavior

Good dietary behaviors, such as non-picky food consumption, having a consistently good appetite, and adherence to a regular timing for meals, will serve to ensure the effectiveness of the dietary treatment.

##### Non-Picky Food Consumption

Caregivers of children who are not picky about the food they consume will face lesser challenges when it comes to the preparation of suitable food.

##### A Consistently Good Appetite

Caregivers of children who maintained a good appetite, even throughout sick days, have less cause for worry about their nutritional status.

##### Eating Behavior

A participant reported that the child under her care is very consistent when it comes to the timing for main meals, and no snacks or other food are taken in between. This facilitated adherence to the prescribed natural protein requirement.

## 4. Discussion

This study employed a qualitative approach of the FGD to investigate the barriers and motivators to dietary adherence among caregivers of AAMD patients undergoing medical and dietetic treatment in Malaysia. With this undertaking, we offer healthcare professionals innovative information on patient needs and strategies aimed at improving the patient’s self-management of their nutritional requirements.

The burden of dietary treatment tops the list of barriers to dietary adherence. We discovered that the majority of caregivers consider the daily diet preparation process complex and arduous. They also perceived the need to comply with dietary restrictions in terms of natural food choices and portions, as well as the strict sick-day regime, as extremely challenging. Previous studies have demonstrated that the dietary treatment significantly affected the quality of life (QoL) of both the patients and their caregivers [39,40,41]. Among the reasons for this poor QoL is the time-consuming and tedious food preparation process, which involves precise weighing and calculations to arrive at an accurate amount of natural protein intake [42]. Additionally, the need to restrict the food portion and the limited availability of suitable food choices also posed problems. It is common knowledge that in European countries, special low-protein foods (SLPFs), such as cereals, bread, energy bar cookies, flour, and rice, are prescribed to patients in order to replace regular protein-containing food. This serves to satisfy the appetite of PKU patients, increase food variety, and improve overall compliance with the restricted dietary regimen [43]. Results from a multinational study revealed that the number of SLPFs available in the prescription list of eight countries ranged between 73 and 276 [44]. In England, the cost of SLPFs, for PKU patients below the age of 18 is fully reimbursed by the government [45,46]. In Malaysia, however, the types and number of SLPFs available are limited, as they need to be imported and probably beyond the reach of those in the low-income bracket, as they are costly [47]. As in Jordan [18], the budget allocated by the government of Malaysia for the treatment of AAMDs is directed mainly towards the procurement of the metabolic formula and a limited variety of SLPFs. In response to this predicament, Malaysian patients incorporate regular low-protein starchy foods such as vermicelli, rice, potato, and flour into their daily diet. However, the protein content in these food products is still significant for patients with AAMDs. As mentioned earlier, caregivers reported that their children were frequently dissatisfied and bored with the limited variety of suitable food products available to them. This issue warrants the attention of the current healthcare service system, as there is an urgent need to introduce low-protein products into the local market at minimal cost so as to render them affordable to all Malaysians afflicted with AAMDs. The results attained through this undertaking are in agreement with those from previous works, which reported that the limited choice of low-protein foods at restaurants and other eating places represents a significant barrier for caregivers and their charges during eating-out sessions [48]

Another barrier identified through this study was the parenting challenges encountered during the care of children with AAMDs. According to the results attained, most caregivers of older children (>7 years old) faced greater challenges in their effort to maintain the natural protein intake within the amount prescribed by healthcare professionals. This finding is in close agreement with those from previous studies, which reported that the decline in dietary compliance and metabolic control accelerates in tandem with the increase in age [46,49]. This circumstance can be attributed to the fact that during the transition from childhood to adolescence, the desire to personally select food products is greatly heightened, as these children would not want their dietary restrictions to get in the way of their relationship with their peers [49,50,51]. Moreover, as an individual’s age increases, the desire to participate in social activities without parental supervision tends to intensify [49]. Caregivers felt stressed during eating-out sessions, as they were reluctant to reveal the dietary requirements of the children, which exposed them to possible discrimination due to their health condition [52,53]. Though this may appear a minor issue, it reflects the Malaysian society’s lack of awareness and knowledge with regard to the issue of metabolic disorders. Caregivers employed a variety of strategies to cope with the stresses posed during eating-out sessions. This includes educating the children under their care on nutrition-related issues, as well as practicing flexibility and moderation, when it comes to their consumption of food high in protein content. According to a previous study, 98% of parents encourage their PKU children to be self-reliant when it comes to their dietary requirements [50]. A well-timed and structured transition, from child-oriented to adult-oriented health care, for any kind of chronic disease, is essential to ensure that children are adequately equipped to assume a lifelong responsibility for their wellbeing [53]. To facilitate flexibility and variety in dietary choices, caregivers reduced the portion of food served. A previous study revealed that a flexible diet was more likely to have a positive impact on diet adherence, as it would reduce the rebellious tendency in children and, consequently, the stress endured by caregivers [54].

The findings from this study also showed that certain dietary behaviors significantly hampered the caregiver’s effort to practice dietary adherence. In terms of food choice, the majority of caregivers stated that their children were choosy about food and reluctant to try new food. This behavior could be attributed to the limited exposure to varied foods in the weaning period [55]. Consequently, this hampers the caregiver’s attempt at providing a varied dietary plan [56]. Interestingly, we discovered that in some patients, feeding problems, such as a poor appetite, lengthy mealtimes, and vomiting, can coexist with the contradictory feeding problem of overeating, which can lead to obesity. Feeding difficulties were observed to be more pronounced among children with urea cycle disorders (UCDs), methylmalonic aciduria (MMA), and propionic aciduria (PA) [56]. These problems appear to be associated with the parental management of feeding behaviors and clinical conditions (such as a neurological impairment), which developed into reduced mobility, abnormal muscle tone, and uncoordinated swallowing [57]. Compared to previous studies carried out among PKU patients [22,23], the feeding problem was not viewed as a barrier to practicing dietary treatment. The disparities in eating behaviors among PKU patients with other AAMD patients are due to their different clinical conditions and the pathophysiology of the different types of disorders.

Generally, this investigation revealed that the majority of caregivers were knowledgeable about the recommended daily natural protein intake requirement and the protein content in food products [50]. Following the early stages of diagnosis, AAMD patients will be referred to a metabolic dietician at the genetic clinic and provided with dietetic counseling during each appointment. The caregivers will be educated on the child’s current protein requirement, a suitable menu, and food selection, which can ensure that the child’s natural protein intake is within the recommended range. Some caregivers remained unclear about the protein content in food and the sick-day regime while lacking food preparation skills. Prior studies have verified the importance of maternal dietary knowledge for improving long-term metabolic control among PKU children [58]. Steps should be taken to further enhance the knowledge of patients and their families regarding the preparation of low-protein diets [59]. Additionally, ‘information about health consequences’ is among the behavior change techniques (BCTs) that can be employed to promote adherence to the treatment plan. The concept here is to deter poor adherence behavior by informing the individual about the price to pay in terms of health for their actions. The majority of participants were convinced that compliance with the treatment diet will have a positive impact on the health of the child. This is among the most important motivators of good dietary adherence [23,24].

Most of the caregivers rated the disease-specific care provided by the inherited metabolic disorder specialists and metabolic dieticians as excellent. Similarly, qualitative work involving caregivers of children with inherited metabolic disorders revealed that they are highly satisfied with the treatment their child received at the specialist metabolic clinic, and the metabolic health care providers were appreciated for their supportive attitude [20,23]. These results are in agreement with those from previous studies, which demonstrated that the support received from family members and social networks is among the most significant motivating factors for adherence to the treatment diet [22]

To the best of our knowledge, this is the first undertaking in Malaysia to explore the barriers and motivators to dietary adherence among the caregivers of AAMD patients. Some of the findings attained through this study are unique and can serve to improve the dietary adherence of AAMD patients in Malaysia. Our use of a virtual meeting platform to conduct the FGD ensured a low dropout rate and a demographically diverse group of participants with a wide variety of AAMDs. However, the use of an online platform is not without drawbacks. Some caregivers were unable to participate in the FGD due to their lack of expertise regarding the use of online platforms, their poor access to an internet connection, or their busy work schedule. Some caregivers were also forced to turn off their video due to poor connectivity to improve the quality and clarity of the audio input. Consequently, this hindered the ability of the moderator to discern the verbal expressions of the participants. Generally, however, although the verbal expressions were not clearly apparent, the virtual FGD facilitated the open sharing of information, opinions, and feedback among the participants regarding the subject matter.

## 5. Conclusions

This study successfully identified several factors, which represented the barriers and motivators to dietary treatment adherence. These factors include knowledge of dietary treatment, eating behaviors, and parenting strategies. To mitigate parental stress due to poor self-control and social stressors, patients should be encouraged to be their own best advocates in the medical decision-making process. The patient with self-care ability should be educated on their condition is lifelong and requires lifelong dietary management. Tailored counseling for individuals, or psychoeducational group interventions, should be made available to patients equipped with the mental capacity to independently care for themselves. This will enable them to enhance the understanding of their medical condition and related treatment needs, as well as execute the required adjustments to their behavior [27,60]. Future research should delve into the perspective of adolescents with AAMDs with regard to the challenges and motivation associated with dietary treatment adherence. This information can facilitate the development of an intervention program tailored to the needs of AAMD patients. Additionally, digital health technologies can be integrated into the intervention program to lessen the food preparation burden of caregivers, as well as enhance the knowledge of patients regarding nutrition and metabolic disorder management. To sum up, caregivers employ a wide range of strategies to render the barriers to creating the successful implementation of a dietary treatment program. The important next step is to develop feasible implementation strategies that could address these barriers and at the same time improve the quality of life of the caregivers, based on further investigations of the local context.

## Figures and Tables

**Table 1:** Focus group discussion questions.

General Questions	Probing Questions
1. Can you share your experience in managing your child’s daily diet?	1. What are the barriers that you faced when managing your child’s daily diet in term of low protein diet and metabolic formula? 2. What are the situations that always trigger non-adherence to dietary treatment? 3. How do you overcome the challenges associated with dietary treatment? 4. What are the factors that continue motivating you to practice the low protein diet although it’s challenging?

**Table 2:** Sociodemographic characteristics of the caregivers of AAMD patients.

Characteristics (*n* = 20)	*n* (%)
Gender Male Female	2 (10%) 18 (90%)
Age of caregiver 31–40 41–50 >50	10 (50%) 9 (45%) 1 (5%)
Age of patients (*n* = 24) 0–3 4–6 7–9 10–12 13–15 16–18	5 (20.8%) 5 (20.8%) 4 (16.7%) 3 (12.5%) 4 (16.7%) 3 (12.5%)
Races Malay Chinese Indian	13 (65%) 6 (30%) 1 (5%)
Educational level Secondary school Diploma/degree	8 (40%) 12 (60%)
Employment Currently working Housewife Retirees	11 (55%) 8 (40%) 1 (5%)
Types of AAMDs of children (*n* = 18) Aminoacidopathies (AAs) Urea cycle disorders (UCDs) Organic acidurias (OAs)	7 (38.8%) 5 (27.8%) 6 (33.3%)

## Data Availability

The data presented in this study are available on request from the corresponding author. The data are not publicly available due to privacy restrictions.

## Supplementary Materials

The following supporting information can be downloaded at: https://www.mdpi.com/article/10.3390/nu14122535/s1, Table S1: Barriers towards dietary adherence; Table S2: Facilitators towards dietary adherence.
