# Supplementary material for: Exploring the Barriers and Motivators to Dietary Adherence among Caregivers of Children with Disorders of Amino Acid Metabolism (AAMDs): A Qualitative Study
Source: Nutrients. 2022 Jun 18;14(12):2535. doi: 10.3390/nu14122535 (PMC9228574; doi:10.3390/nu14122535)
Supplement: Supplementary file 1 [file nutrients-14-02535-s001.zip › nutrients-1750657-supplementary.pdf]

Table S1. Barriers towards dietary adherence

Themes	Subthemes	Quotations
Burden of dietary treatment	Tedious food preparation	"… the food is getting cold after a long time of weighing and calculating…and I need to heat up again for my child to eat…" (007, Chinese, Female, 41 years old)<br>"We have to calculate so they do not exceed their required protein, neither should we give them below what is required because they need the protein in order to grow, so that is the difficult part… the problem is calculating it a little bit for me la, for old people like me grandmother (laughing), is a bit difficult to calculate it…" (013a, Chinese, 73 years old)<br>"… it's too challenging for someone new to it like me… we need to calculate the total intake… need to take care of the metabolic formula … need to think about what to fed her…". (010, Malay, 40 years old)<br>"… I use the low protein flour given by the hospital to make yellow noodles…I have failed making it 2-3 times…just threw away…it's quite sensitive…cannot put excessive water… cannot put lesser water…like that…" (006, Malay, 42 years old)
	Struggle to find food when out of the home	"… travelling is quite hard, hard to predict the protein, you can say, you can avoid meat but they will be diary so that will be one of the biggest challenges…" (002b, Chinese, 40 years old)<br>"…she wanted to buy the food in canteen…but there is no diet for her…if we request…canteen won't necessarily will follow what we say… as only one student like her required the diet…they can't prepare it…" (003, Malay, 43 years old)<br>"…when we admitted to hospital…usually they won't prepare the diet we requested…that's the challenge…every time admitted… we need to quarrel with the staff in the hospital…we requested porridge…but they give us rice …" (004, Malay, 43 years old)
	Limited food portion	"…like potato, he can eat, but he cannot eat in a large quantity, … if he ate the potato in a large amount, we will know the changes (symptoms)…" (012, Malay, 33 years old)<br>"… the challenge is…he does not eat enough…he wants to eat more…just half cup of rice is not sufficient for him…" (004, Malay, 43 years old)<br>"…she is quite thin, like does not receive enough nutrient…if she ate…rice was a little bit and limited…not much she can eat…she can't gain weight…" (018, Malay, 33 years old)
	Limited food choices	"…like our kids' birthday, it is quite hard to get them a how to say, low protein cake that they can enjoy…" (002b, Chinese, 40 years old)<br>"…sometimes only pasta is provided…she was bored with it…like one of the challenges…not much variety of low protein food…" (018, Malay, 33 years old)

	Contradicting nutrients	"When it comes to low protein food, mostly are fibre and some vitamin and stuff, but also very high in carb and sugar... when we study the content, mostly are really a lot of sugar, so I think … the biggest concern is in terms of this nutritional value…" (002b, Chinese, 40 years old)
	Challenges during sick day	"When he was sick, he can't eat anything… that's very painful… but sometimes he requested for rice also… he can't eat on that day…he can only drink milk on that day…that's very challenging for me la…" (012, Malay, 33 years old)
Parenting challenges	Social Stressors	"… he started to see all his cousins eating all kinds of food… he also started to request all kinds of food… he will throw tantrum when he wanted to eat chicken and many more…a while la, he will throw the tantrum…" (001, Indian, 42 years old)<br>"… she saw a variety of menu in the school canteen, she felt like eating everything…" (003, Malay, 43 years old)<br>"…when he was small, we as parents can arrange for them, can manage (what to give him to eat) …, but when he has grown up, there will be certainly a problem…" (012, Malay, 33 years old)
	Poor self-control	"… during MCO my children stayed at home and I went to work…left three of them at home…I prepared fried rice for them to eat…they might eat the rice exceeding the recommended portion…and sometimes forgetting to take their metabolic formula…" (005, Malay, 44 years old)<br>"…when she was a small kid, she always stoles food at home…she is not in front of us…if we can supervise her than it's ok… she's not within our supervision…it's very challenging to supervise her…very challenging…" (003, Malay, 43 years old)
	Parental stress	"When I was trying to look for kindergarten for him, nobody dares to take him in, because his diet like that, like they were worried they will give him high protein food." (002a, Chinese, 38 years old)<br>"We sometimes will really ask him to eat more… just try not to when he said full, we just let him be… because sometimes… we do notice like when he wants some treats, so he don't have the patience to finish his food or his milk, than he will be like forcing himself to drink quickly or he will pretend sick, you know like, I am too full, I don't want to eat…sometimes we just kind and force it for him to drink we know he needs the milk, but there are times where he really… full and … can't take it and vomit…" (002b, Chinese, 40 years old)<br>"…when people around look at us…they were like…why…like…they don't understand us…I felt lazy to explain to them…so I will just tell my children are not interested in eating (fish and chicken) …if we ate at KFC or MCD, I will just order fries for them…but people keep looking at us…why our children do not eat the chicken…" (005, Malay, 44 years old)<br>"… I also feel like MSUD is not very well known, at least in Malaysia and most countries… we have a lot of awareness...like AIDS, some other disease, diabetes and so on, but I think when come to metabolic, is really something a lot people doesn't know…" (002b, Chinese, 40 years old)

	Impaired self-care skills	… my son is still not good at taking care of his own, if he went to the toilet, he also cannot clean himself well… we have to ask the teacher to prepare the milk… and ask the teacher to give him to drink… need to make sure he finished drinking the milk…it's a bit troublesome la…" (011, Chinese, 36 years old)
Limited knowledge related to dietary management	Uncertainty about food	"Sometimes when we have a fruit or a vegetable which is not inside the chart, we don't know what to do, so we just keep it…" (013a, Chinese, 73 years old)<br>"Normally because we are scared that we might exceed the protein level, so we ended up in always giving less…" (013a, Chinese, 73 years old)<br>"… she fell sick as insides the (sandwich) consist of egg, she thought she can eat the bread, but (ingredients) inside the sandwich she can't eat…" (003, Malay, 43 years old)
	Uncertainty about sick-day regime	…if really when metabolic crisis happens... really yes, there is no step by step how to do it… (002b, Chinese, 40 years old)<br>"… when he was sick, I really… (hand gesture rubbing at chin) no idea what should do… he doesn't want to eat anything… so unable to meet the protein intake prescribed by doctor to him…" (011, Chinese,36 years old)
	Limited knowledge in food preparation	"… sometimes parents, working parents we also don't have idea how to cook everything…" … (001, Indian, 42 years old)
Diet and Dietary Behaviour	Food Choice	… not interested in taking bread, spaghetti, … and all the low protein products… gave them before but they don't like… usually they only eat Malay version rice…" (005, Malay, 44 years old)<br>"… he is choosy a bit, he doesn't want to eat this and that…, if he wants to eat, want plain curry with rice only…it's very hard to get my son to eat vegetables… (001, Indian, 42 years old)<br>"… his routine is about eating rice with potato, he only wants that, he doesn't want to eat other food…" (004, Malay, 43 years old)<br>"…the challenge is…my child refused to eat what I had prepared for her, I had weighed the food for one day…she did not want to eat…, we need to create new idea for her…" (009, Malay, 37 years old)
	Eating Behaviour	"Sometimes when he over eat he always vomit everything out, that's my challenge… sometimes, after eat, not long, after he had drink milk, he will vomit everything out…" (002a, Chinese, 38 years old)<br>"…my son is not interested in eating, it is a bit troublesome…he eats very slowly, for example he needs to take 1-2 hours to finish one meal, if more ok he will take 1 hour…sometimes when he eats faster… needs approximately 40 minutes for one serving of nasi lemak, but he is not good in self-feeding, that's why he needs to take a longer time…, he will need to pick up the vegetables one by one (hand gesture showing picking up the food one by one) (007, Chinese, 41 years old)

		"… the problem is she eats a lot …, so we need to control her… if not, she will eat like… uncontrollable… (emphasize tone, frowning) … her body weight can be hard to control, especially during this MCO session, she will stay at home and eat … she preferred to eat heavy meals… (017, Malay 39 years old)
Challenges in delivery healthcare system	Limited supply of low protein food	"…I had to buy (low protein) food for my child on my own, as the food given by the hospital is not much…" (006, Malay. 44 years old)<br>"… the low protein flour… sometimes we get, but sometimes there is no stock…, so that's one of the problems also…" (018, Malay, 33 years old)
	Lack of communication between healthcare providers	"… I called the dietitian in HKL to ask her to inform the dietitian in the hospital he will admit into it… want to tell them … the food service department in the hospital to prepare the special diet for him… but we never get the diet that we requested…" (004, Malay, 43 years old)
	Long waiting time	"… hospital you know arh…, sometimes it keeps ringing and ringing. nobody picks up the phone, so we can panic what to do…" (004, Malay, 43 years old)

Table S2. Facilitators towards dietary adherence

Themes	Subthemes	Quotes
Good Knowledge of inherited metabolic disorders	Good knowledge of dietary treatment	"… if she wants to eat (extra food) during breakfast, normally I won't give other food, I will give her food which is lower in protein content such as watermelon… (008, Malay, 32 years old)<br>"… for her diet calculation… I think I do not need to refer to the book given by the dietitian… I can estimate…how much needed by her in one day… what is needed… the measurement for potato… I have been so used to measuring it…I can estimate how many grams (of protein) of one potato…it had been one year ago since I started to learn how to calculate my child's diet... all these things were inside my mind now… (010, Malay, 36 years old).<br>"… her daily protein requirement is only limited to 5g only… I will cook for her 3g of protein during lunch, for dinner I will cook for here the remaining 2g of protein …" (018, Malay, 33 years old).<br>"… for 6g of protein… I will divide it into three meals…, for example rice, half cup equals to 1g of protein. For breakfast, lunch and dinner, we will give him only half cup of rice… the remaining we will give vegetables …potato or carrot…quarter of potato each meal… adding up the vegetables with rice, he will obtain 1.5g of protein… the remaining we will give him biscuits…we will calculate the biscuits…for example 3 pieces equal to 1g protein… so we will estimate 3 pieces for one day…." (018, Malay, 33 years old).<br>"… when she had some sick symptoms… I will stop her from eating…, I will straight away prepare the milk (metabolic formula), and will increase the amount of valine and isoleucine as well as increase the amount of milk… I will check her urine…if her urine is ok…or she did not show symptoms anymore…I will ask her if she is ok…if everything ok, I will start feeding her as usual…that's my experience taking care of her… (008, Malay, 32 years old).
	Creativity in meal preparation	"…if she wanted to eat cookies, I will use corn starch to bake cookies for her, because corn starch can be a lot…" (008, Malay, 32 years old)<br>"…if she wanted to eat Mee Hoon, Mee Hoon is high in protein…, one day maybe she will only eat Mee Hoon for breakfast and lunch, dinner she will eat special low protein food … if she ate low protein rice…let's say… or macaroni… spaghetti low protein, she can eat quite a lot at once…because the protein content is very minimal…" (006, Malay, 42 years old)<br>"…if she doesn't want to eat rice…I will replace rice with pumpkin…we can boil the pumpkin, and makes it into finger foods, mixed with floor and fried…sometimes I also made laksa for her…because when laksa is boiled…the protein content is very low…when our children don't want to eat rice, we can change to laksa" (010, Malay, 42 years old)
	Health concern	"…if he overate… we will suffer…he will suffer…need to admit to hospital…" (001, Indian, 42 years old)

		"…for my child's disease…when there was an excessive protein intake…it will affect the movement of her brain…she might become a disabled person…cannot walk…glutaric aciduria will affect the body movement…" (009, Malay, 37 years old)
		"…what I learned about MSUD is if you have too much protein, and then you get like drunk effect and then it will spoil your nervous system, spoil your brain and spoil everything… (002b, Chinese, 40 years old)<br>"…we want to have an accurate amount of protein intake for him, so that he can have enough to develop his leg muscle, in order to future maybe he can walk…" (013b, Chinese, 41 years old)<br>"…he needs the formula six times per day, or 5 times, if he only drinks once, he will have no energy…he cannot walk, we will feel too sad for him…" (012, Malay, 33 years old)
Parental coping strategies	Flexibility and moderation	"…sometimes I also give her laksa gravy, but just for the sake of satisfying her…I will add in warm water… let her to taste the salty… I also give her Milo… accordingly that is a prohibited food for her… as it contains high protein…but I just made for her just to let her taste…so I will reduce the concentration…just for cheating her… (017, Malay, 37 years old)<br>"…it's just kind like give and take, I mean of course it won't be 100% accurate, in facts certain kuih that we know is usually made from rice flour for instance, so that is usually quite safe because based on rice arh... protein is not very much but there are certain they made with other flour, high protein flour…, when comes to case like that, we have doubts, usually we cut half and so far, no issue with his protein level…." (002b, Chinese, 40 years old).<br>"…we will replace it with some foods she was allowed to eat… so she won't be aggressive and angry when she cannot get something she wanted…" (010, Malay, 36 years old).<br>"…Kuih, ya, I won't give much la, just give half for one kuih only, let say he eat a lot, I just cut the infant formula, the milk. If I have to put one scoop, I only put half scoop for the next feeding…" (001, Indian, 42 years old)<br>"…let's say one piece of banana is equal to 1g of protein…what we have prepared for her…either vegetables or rice…when she did not finish… we can estimate like…0.5g of protein not yet finished…so we will top-up with half gram of protein from banana…for example la…last option I will top-up with milk… (009, Malay, 37 years old)
	Self-prepared food when out	"When we go to places with limited choices, I (do) brought small rice cooker to use to carry everywhere we went... the pasta provided by the genetic clinic, I usually bring that everywhere I go, in case he really cannot find other food, just cook the pasta and some tomato sauce, pasta sauce…" (002a, Chinese, 37 years old)<br>"…when we stay overnight… always I will order white rice…my 2nd son likes to eat fried onions, so no problem for him… I will prepare fried onions for him from home…I will order white rice and he will eat the white rice with fried onion…" (005, Malay, 43 years old)

		"…she will bring one cooler bag and one lunch box to school…that's her routine when going school…her teacher will keep the milk inside the fridge…, and she will eat the food and drink the milk during recess time…" (006, Malay, 42 years old)
	Nutrition Education	"…I will tell him, if you eat more, you will need to be admitted… you will fall sick, you will vomit, do you want to be admitted into hospital…" (001, Indian, 42 years old)<br>"…when she came back (from school), I will write it down…what she can eat…and send all the pictures to her…what she can eat a little bit…what she cannot eat at all…those food that she cannot eat I will put a cross on it…" (003, Malay, 43 years old)<br>"…I will bring him to KFC…I will tell him what he can eat…what he cannot eat…he can eat the fries…we have to bring him together…there will be time when he will go out friends… I am worried…so it's better to educate him since young…" (012, Malay, 33 years old)
	Good self-care	"… I asked her to prepare her milk in school by her own, she can prepare by her own, she will prepare the Ketonex together with valine and isoleucine…" (003, Malay, 43 years old)<br>"…if she bought (food in canteen), she will buy the sweet kuih only…such as kuih lapis…if she wanted to eat Karipap, she will throw away all the ingredients inside, sometimes insides the Karipap contains of anchovies and chicken…" (003, Malay, 43 years old)<br>"…last time someone offered her a food to eat… she will say "cannot", "my mom won't allow… "we will fall sick if we eat", she will know it…" (006, Malay, 42 years old)
Social Coping	Schools	"…when I put her under special education school, one teacher can control five students…so the teacher can supervise our child, what out child can eat…she will bring it (this issue) to canteen one week before…" (003, Malay, 43 years old)<br>"…I will tell the teacher…she is not allowed to give any food to my child…except what I have prepared for her…so far no problem…the teacher will follow what I said…" (006, Malay, 42 years old)<br>"…when I didn't accompany him to school, I need to ask the teacher to prepare the milk…need to ensure he finished the milk…I give the milk to the teacher…teacher will give him to drink…" (012, Malay, 33 years old)
	Healthcare system	"I really follow the book…the book given by the hospital…the book is very useful…" (012, Malay, 33 years old)<br>"…the calculation needed to be done on his metabolic formula ...I have a trend…every time when he was unwell …the first thing I will do is to call the doctor" (011, Chinese, 37 years old)<br>"…we attend a dietitian session whereby they will recalculate for us and check with us again what we had been giving the twins…" (013a, Chinese, 73 years old)

		"…one of the methods for me to help the genetic department to achieve their objectives…" (004, Malay, 43 years old)
	Family Members	"…I left him with his grandmother, so his grandmother will monitor la…I tell his grandmother…how much he can eat…so his grandmother will give him that amount only…:" (004, Malay, 43 years old)
		"…in school, she had her brother with her…her brother will help her…observe her…whether she eats ok or not…" (008, Malay, 33 years old)
		"…I stopped working for my child…most of the food I will self-prepare at home…" (006, Malay, 42 years old)
	Other caregivers	"…when I asked other parents meticulously… sometimes when she admitted to the hospital we will ask…how they take care of their children, how they feed their children, how to know the child has overeaten… what are the symptoms…" (006, Malay, 42 years old)
Diet and Dietary Behaviour	Unfussy eater	"… no problems with vegetables, she will eat all…" (015, Malay, 40 years old)<br>"…like my child…even I just cooked the laksa with soy sauce, she will still eat, she won't reject it…" (010, Malay, 36 years old).
	Good Appetite	"…her appetite is good, other people said when children fall sick, they will have poor appetite, but she is still consistent to finish her food…she still finished all the milk, she also ate a lot … so I am not worried about her…" (010, Malay, 36 years old).
	Eating behaviour	"…she only eats three times per day… morning, 7 am, 12 pm, afternoon for lunch, and evening 5pm for dinner…so she does not have other food besides these times…she didn't have extra food like 8pm for biscuits, she does not have supper" (006, Malay, 43 years old).
